# The Parameter-Optimized Recursive Sliding Variational Mode Decomposition Algorithm and Its Application in Sensor Signal Processing

**DOI:** 10.3390/s25061944

**Published:** 2025-03-20

**Authors:** Yunyi Liu, Wenjun He, Tao Pan, Shuxian Qin, Zhaokai Ruan, Xiangcheng Li

**Affiliations:** 1 The Guangxi Key Laboratory of Multimedia Communications and Network Technology, Guangxi University, Nanning 530004, China; liuyunyi@gxu.edu.cn; 2 School of Computer and Electronic Information, Guangxi University, Nanning 530004, China; 2213391002@st.gxu.edu.cn (W.H.); 2313392017@st.gxu.edu.cn (T.P.); 2113391109@st.gxu.edu.cn (S.Q.); ruanzhaokai@st.gxu.edu.cn (Z.R.)

**Keywords:** variational mode decomposition, recursive sliding, parameter optimization, IMU signal, denoising

## Abstract

In industrial polishing, the sensor on the polishing motor needs to extract accurate signals in real time. Due to the insufficient real-time performance of Variational Mode Decomposition (VMD) for signal extraction, some studies have proposed the Recursive Sliding Variational Mode Decomposition (RSVMD) algorithm to address this limitation. However, RSVMD can exhibit unstable performance in strong-interference scenarios. To suppress this phenomenon, a Parameter-Optimized Recursive Sliding Variational Mode Decomposition (PO-RSVMD) algorithm is proposed. The PO-RSVMD algorithm optimizes RSVMD in the following two ways: First, an iterative termination condition based on modal component error mutation judgment is introduced to prevent over-decomposition. Second, a rate learning factor is introduced to automatically adjust the initial center frequency of the current window to reduce errors. Through simulation experiments with signals with different signal-to-noise ratios (SNR), it is found that as the SNR increases from 0 dB to 17 dB, the PO-RSVMD algorithm accelerates the iteration time by at least 53% compared to VMD and RSVMD; the number of iterations decreases by at least 57%; and the RMSE is reduced by 35% compared to the other two algorithms. Furthermore, when applying the PO-RSVMD algorithm and the RSVMD algorithm to the Inertial Measurement Unit (IMU) for measuring signal extraction performance under strong interference conditions after the polishing motor starts, the average iteration time and number of iterations of PO-RSVMD are significantly lower than those of RSVMD, demonstrating its capability for rapid signal extraction. Moreover, the average RMSE values of the two algorithms are very close, verifying the high real-time performance and stability of PO-RSVMD in practical applications.

## 1. Introduction

Currently, wavelet transform and modal decomposition are the mainstream signal denoising techniques. Wavelet transform is a signal processing technique based on wavelet analysis. It performs multi-scale refinement analysis of the signal through scaling and shifting operations, effectively handling the signal. Zhang et al. [1] proposed an improved wavelet denoising algorithm, which uses the concept of adaptive weighted averaging to determine the boundary between noise and signal components, and reconstructs the filtered signal accordingly. However, the choice of wavelet basis heavily relies on human experience, and improper selection can affect the decomposition performance of the signal.

Empirical Mode Decomposition (EMD) is one of the mainstream modal decomposition algorithms. When applying EMD to remove signal noise, the signal is decomposed into several intrinsic mode functions (IMF), and the selection criteria for noise IMF are crucial. Many researchers have studied various correlation-based methods as noise determination criteria. For example, in reference [2], the Bhattacharyya distance and the variance of the autocorrelation function are used in a two-step process to filter out noise components. Shi et al. [3] distinguish noise components from signal components based on the correlation coefficient between each component and the original signal. Reference [4] uses the Hellinger distance to filter out noise components, then extracts valid signal components using the autocorrelation function, and finally reconstructs compensation errors by combining the signal components with the residual. Wang et al. [5] remove high-frequency components, then use the TFPF algorithm to extract useful signals from the intermediate-frequency components, and finally merge the extracted signal with the low-frequency components to reconstruct the original signal. These algorithms achieve signal denoising but carry the risk of losing useful signals, leading to bias in the reconstructed signal. Meanwhile, traditional EMD cannot decompose a signal if there are insufficient extreme points, which can result in spectral overlap. To solve this issue, some studies have attempted to improve the EMD algorithm. For example, an enhanced method effectively separates measurement noise in drift signals using a combined EMD approach [6]. This improvement significantly mitigates the mode mixing problem of EMD but does not fundamentally eliminate the white noise introduced during the decomposition process.

Variational Mode Decomposition (VMD) [7], as an advanced signal processing technique, is particularly suitable for the analysis and processing of nonlinear and non-stationary signals. When using the VMD algorithm for denoising, the decomposition parameters and noise separation criteria need to be pre-set. The precise separation of signal and noise can only be effectively achieved when the decomposition level, penalty factor, and other parameters are matched with the time-frequency characteristics of the signal to be processed. To find the optimal decomposition level and penalty factor for a signal, Wang et al. [8] used a differential evolution algorithm to search for the optimal combination of decomposition level and penalty factor in VMD. Other studies have introduced population-based heuristic optimization algorithms into the VMD parameter pre-setting stage, such as the Beetle Antennae Search Algorithm [9], but these methods have higher computational complexity and certain randomness. To suppress truncation and endpoint effects during signal decomposition, reference [10] used a relatively simple and direct mathematical statistical method. It employed a triangular waveform matching approach to search the entire signal and find the waveform most compatible with the endpoints, and then used a grid search method based on mutual information entropy criteria to select the optimal VMD parameters for the extended signal.

VMD has demonstrated superior decomposition performance in modal decomposition, making it widely applicable in various fields. In the sensor domain, Inertial Measurement Units (IMUs) are commonly used in applications such as autonomous driving [11], navigation [12], robotics [13], and industrial polishing [14]. IMUs are capable of capturing and transmitting key kinematic information such as acceleration and angular velocity in real time, which is crucial for achieving precise control. However, in practical applications, IMU signals are often inevitably affected by noise, vibrations, and other interference factors. These disturbances not only reduce the accuracy of the signals but also directly impact subsequent data processing and analysis. In industrial polishing operations, it is necessary to ensure the precision of both the polishing process and the real-time performance, which calls for improved IMU signal denoising capabilities. We applied VMD to denoise IMU signals under industrial polishing conditions. However, the iterative optimization mechanism of VMD limits its decomposition efficiency to some extent, making it less effective in handling large-scale signals or signals with high real-time requirements.

In reference [15], the authors proposed a novel method, the Recursive Sliding Variational Mode Decomposition (RSVMD) algorithm. This method replaces the original Fourier transform of the model with sliding discrete Fourier transform. To enhance decomposition efficiency while ensuring accuracy, the method incorporates prior knowledge from previous decompositions to set the initial decomposition parameters for the current window and imposes stricter constraints on the iterative process. RSVMD significantly improves decomposition efficiency while maintaining accuracy, making it more suitable for applications with high real-time requirements. However, through simulation experiments, we found that when processing signals with strong interference components, RSVMD exhibits unstable performance, which manifests as two main phenomena: First, there is a dramatic increase in iteration time and iteration count; second, the center frequency is incorrectly initialized after the window data are updated. These two phenomena lead to a decrease in signal decomposition efficiency and insufficient output accuracy.

While the general signal decomposition capability of VMD-based methods is well established, the primary focus of this work is to address the real-time performance limitations of RSVMD. Specifically, we aim to improve the computational efficiency of RSVMD in real-world applications, such as IMU signal processing in industrial polishing, where real-time performance is critical. Although the decomposition accuracy of RSVMD is satisfactory, its computational load and iteration time are often prohibitive in scenarios requiring high real-time performance. Therefore, the RSVMD algorithm needs to undergo parameter optimization, i.e., PO-RSVMD. To address the two unstable performance phenomena, an iterative termination condition based on modal component error mutation judgment is introduced to avoid over-decomposition, which can lead to a significant increase in computational load and a sharp decline in performance. At the same time, considering that performance degradation may lead to errors in the center frequency band, a rate learning factor is introduced to reduce the center frequency error. The algorithm combines the current center frequency with the previous window’s center frequency to obtain the new center frequency. Since the trend of iteration speed and algorithm performance is consistent, the rate learning factor is automatically adjusted based on the change in iteration time to maintain the initial center frequency. Then, through signal decomposition simulation experiments, the performance of the VMD, RSVMD, and PO-RSVMD algorithms is compared. Additionally, we attempt to apply the PO-RSVMD algorithm to the denoising of IMU angular velocity signals during polishing, with the expectation that PO-RSVMD can quickly and accurately complete the decomposition of angular velocity signals and the removal of interference components.

## 2. Algorithm Principles

### 2.1. Algorithm Fundamentals

Variational Mode Decomposition (VMD) is a completely non-recursive method of signal decomposition that works by decomposing a signal into a series of intrinsic mode functions (IMFs) with specific center frequencies and finite bandwidths. Given an input signal f(t), the expression of the *k*-th IMFs obtained by VMD decomposition is shown in Formula (1).

(1) $$v_{k}\left(t\right)=A_{k}\left(t\right)\cos\left(\varphi_{k}\left(t\right)\right)$$

where $A_{k}\left(t\right)$ represents the instantaneous amplitude of the corresponding mode component, and $\varphi_{k}\left(t\right)$ is the instantaneous phase. The instantaneous center frequency can be obtained by differentiating the instantaneous phase.

The core idea of VMD algorithm is to reconstruct the constrained variational model and obtain the optimal solution. The resulting construction of the constrained variational mode is shown in Formulas (2) and (3).

(2) $$\min_{\left\{v_{k}\right\},\left\{\omega_{k}\right\}}\left(\sum_{k=1}^{K}{∥\partial_{t}\left[\left(\delta \left(t\right)+\frac{j}{\pi t}\right)*v_{k}\left(t\right)\right]e^{-j\omega_{k}t}∥}_{2}^{2}\right)$$

(3) $$s.t.\sum_{k=1}^{K}v_{k}\left(t\right)=f\left(t\right)$$

where $v_{k}$ is the set of all modes, $\omega_{k}$ is the set of center frequencies, and s.t. denotes the constraint.

In order to render the constrained variational model no longer constrained, the quadratic penalty term α and the Lagrange multiplier λ are utilized to address the reconstruction. Thus the obtained augmented Lagrange expression is shown in Formula (4).

(4) $$\begin{array}{c} L\left(\left\{v_{k}\right\},\left\{\omega_{k}\right\},\lambda \right)=\alpha \sum_{k=1}^{K}∥\partial_{t}\left[\left(\delta \left(t\right)+\frac{j}{\pi t}\right)*v_{k}\left(t\right)\right]{e^{-j\omega_{k}t}∥}_{2}^{2}\\ +{∥f\left(t\right)-\sum_{k=1}^{K}v_{k}\left(t\right)∥}_{2}^{2}+\langle \lambda \left(t\right),f\left(t\right)-\sum_{k=1}^{K}v_{k}\left(t\right)\rangle \end{array}$$

We use the Alternate Direction Method of Multipliers (ADMM) [16,17] to obtain the optimal solution of the variational mode iteratively, and the expression of the modal components $v_{k}^{n+1}$ is shown in Formula (5).

(5) $$v_{k}^{n+1}=agr\min_{v_{k}\in X}\left(\alpha \sum_{k=1}^{K}{∥\partial_{t}\left[\left(\delta \left(t\right)+\frac{j}{\pi t}\right)*v_{k}\left(t\right)\right]e^{-j\omega_{k}^{n+1}t}∥}_{2}^{2}+{∥f\left(t\right)-\sum_{i\neq k}v_{i}^{n+1}\left(t\right)+\frac{\lambda (t)}{2}∥}_{2}^{2}\right)$$

where *n* is the number of iterations.

The principle of Sliding Discrete Fourier Transform (SDFT) [18,19] is to use the sliding window to dynamically add new data and remove old data, and perform Discrete Fourier Transform (DFT) to eliminate the redundant calculation. The expression of the single-sample sliding discrete Fourier transform is shown in Formula (6).

(6) $$X_{1}\left(k\right)=\left[X_{0}\left(k\right)-x\left(0\right)+x\left(N\right)\right]e^{j\frac{2\pi k}{N}}$$

When the single sample point is generalized to multiple sample points, that is, when the step size is *s*, the corresponding expression of sliding window SDFT with multiple sample points is shown in Formula (7).

(7) $$X_{s}\left(k\right)=X_{0}\left(k\right)e^{j\frac{2\pi ks}{N}}+\sum_{p=0}^{L-1}\left[x\left(p+N\right)-x\left(p\right)\right]W_{N}^{k(p+1)}$$

where $X_{s}\left(k\right)$ is the spectrum of in the current window, x(p+N) is the new sampled data added to the current window, and x(p) is the old sampled data to be removed from the current window; the complex exponential $W_{N}^{k(p+1)}$ is a rotational factor influenced by the shifting parameter *k*.

We use SDFT to convert Formula (5) into the frequency domain, and the optimized mode components are shown in Formula (8).

(8) $$v_{k}^{n+1}\left(\omega \right)=\frac{SDFT\left(\omega \right)-\sum_{i\neq k}{\overset{⌢}{v}}_{i}^{n+1}\left(\omega \right)+\left(\overset{⌢}{\lambda }\left(\omega \right)/2\right)}{1+2\alpha {\left(\omega -\omega_{k}\right)}^{2}}$$

where SDFT(ω) is the spectrum data obtained by SDFT calculation.

Similarly, the updated center frequency after iteration $\omega_{k}^{n+1}$ is shown in Formula (9).

(9) $$\omega_{k}^{n+1}\approx \frac{\sum \omega {\left|{\overset{⌢}{v}}_{k}\left(\omega \right)\right|}^{2}}{\sum {\left|{\overset{⌢}{v}}_{k}\left(\omega \right)\right|}^{2}}$$

### 2.2. Parameter-Optimized RSVMD Algorithm

The PO-RSVMD algorithm is designed to address the real-time performance limitations of RSVMD, particularly in scenarios with strong interference. While RSVMD demonstrates satisfactory decomposition accuracy, its computational load and iteration time can be prohibitive in real-time applications. The primary contribution of PO-RSVMD lies in its ability to reduce computational complexity while maintaining comparable decomposition accuracy, rather than extending its applicability to a broader range of signal types. Although the algorithm’s performance in handling arbitrary amplitude or frequency modulation signals is an important direction for future research, the current study focuses on optimizing RSVMD for real-time applications. The optimization strategy of RSVMD will be described in detail below.

#### 2.2.1. Supplementary Constraints for Convergence Conditions

The two iteration termination conditions of VMD mode are the maximum number of iterations and the minimum convergence error, and the updating process for iteration is terminated when one of the conditions above is met. In order to avoid over-decomposition in the iterative update process, the relative error judgment is added into the original error judgment condition. When the error is greater than the preset accuracy, the iteration is terminated.

(10) $$\sum_{k}\frac{{∥v_{k}^{n+1}\left(\omega \right)-v_{k}^{n}\left(\omega \right)∥}_{2}^{2}}{{∥v_{k}^{n}\left(\omega \right)∥}_{2}^{2}}<\varepsilon_{r}$$

(11) $$\sum_{k}{∥v_{k}^{n+1}\left(\omega \right)-v_{k}^{n}\left(\omega \right)∥}_{2}^{2}<\varepsilon_{a}$$

where $\varepsilon_{r}$ represents the relative error, and $\varepsilon_{a}$ represents the absolute error.

Although the algorithm obtains a relatively ideal result in the iteration process, it is possible that the iteration process will continue until the maximum number of iterations is reached, resulting in a certain error between the final result and the theoretical value. Therefore, the iterative convergence condition requires further supplementary constraints to prevent the RSVMD mode from falling into over-decomposition. Over-decomposition is a situation where an excessive operation volume is generated due to deterioration in the performance of the algorithm, which makes the signal decomposition accuracy become lower. According to the literature on the original VMD algorithm, VMD uses the ADMM solution. Due to the convergence of ADMM, the original residual $r^{n}$ will gradually approach 0. The VMD solution is based on constraints such as Formula (3) above. The original residual is represented by Formula (12).

(12) $$r^{n}=f\left(t\right)-\sum_{k=1}^{K}v_{k}^{n}\left(t\right)$$

According to the ADMM update rules, the number of updates to Lagrangian multipliers is shown in Formula (13).

(13) $$\lambda^{n+1}=\lambda^{n}+\alpha \left(f\left(t\right)-\sum_{k=1}^{K}v_{k}^{n+1}\left(t\right)\right)$$

When performing the iterative solution, the dual residual of ADMM is represented by Formula (14).

(14) $$s^{n}=\lambda^{n+1}-\lambda^{n}=\alpha \left(f\left(t\right)-\sum_{k=1}^{K}v_{k}^{n+1}\left(t\right)\right)$$

According to the constraints, the dual residual can be expressed by Formula (15).

(15) $$s^{n}=\alpha \sum_{k=1}^{K}\left(v_{k}^{n+1}\left(t\right)-v_{k}^{n}\left(t\right)\right)$$

According to the convergence of ADMM, the original residual $r^{n}$ will gradually approach 0, so the dual residual $s^{n}$ will gradually approach 0. For the relative error $\varepsilon_{r}$ and absolute error $\varepsilon_{a}$ in the iterative exit condition, since the objective function is monotonically decreasing and has a lower bound, its residuals also converge to 0. This is proven as follows.

In each iteration, updating of the modal function $v_{k}\left(t\right)$ and the center frequency $\omega_{k}$ is achieved by minimizing the objective function. Because the objective function is monotonically decreasing, the change in the modal function and the center frequency in each iteration will gradually decrease; otherwise, the value of the objective function cannot continue to decrease. Specifically, if the change in the modal function $v_{k}\left(t\right)$ is large, the objective function will be significantly reduced. As the iteration progresses, the modal function and the center frequency gradually approach the optimal value, the amount of change will decrease, and the decrease in the objective function value will gradually decrease. When the number of iterations n→∞, the objective function converges to the local minimum value, and the change in the modal function $v_{k}\left(t\right)$ and the center frequency $\omega_{k}$ is close to 0 ${∥v_{k}^{n+1}\left(t\right)-v_{k}^{n}\left(t\right)∥}_{2}\to 0$, $\left|\omega_{k}^{n+1}-\omega_{k}^{n}\right|\to 0$. Therefore, during the iteration process, especially when the iteration error converges to a smaller value, the larger error can clearly indicate that the system iteration is in an abnormal situation and the iteration should stop. Therefore, based on the monotonic judgment of the above error changes, the supplementary constraints are shown in Formulas (16) and (17).

(16) $$k_{r}=\frac{\varepsilon_{r}^{n}-\varepsilon_{r}^{n-1}}{n-(n-1)}=\varepsilon_{r}^{n}-\varepsilon_{r}^{n-1}<0$$

(17) $$k_{a}=\frac{\varepsilon_{a}^{n}-\varepsilon_{a}^{n-1}}{n-(n-1)}=\varepsilon_{a}^{n}-\varepsilon_{a}^{n-1}<0$$

where *n* is the number of current iterations, $k_{r}$ is the slope of the relative error, $\varepsilon_{r}^{n}$ and $\varepsilon_{r}^{n-1}$ are the relative errors of two adjacent iterations, $k_{a}$ is the slope of the absolute error, and $\varepsilon_{a}^{n}$ and $\varepsilon_{a}^{n-1}$ are the absolute errors of two adjacent iterations. When the above supplementary constraint conditions are not satisfied, the iterative optimization process is terminated immediately.

#### 2.2.2. Optimization of Center Frequency and Modal Components

In order to make the algorithm converge quickly, using the rate factor *a* to fuse the initial parameters and result parameters in the previous window proportionally. This can ensure the reasonable utilization of the data in the current window and improve the decomposition efficiency. From this, the current initial center frequency is constructed as shown in Formula (18).

(18) $${\left\{\omega_{k}^{0}\right\}}_{\mathrm{current}\;}=\left(1-a\right){\left\{\omega_{k}^{0}\right\}}_{\mathrm{last}\;}+a{\left\{\omega_{k}^{e}\right\}}_{\mathrm{last}\;}$$

where ${\left\{\omega_{k}^{0}\right\}}_{current}$ is the initial center frequency of the current window, ${\left\{\omega_{k}^{0}\right\}}_{last}$ is the initial center frequency of the previous sliding window, and ${\left\{\omega_{k}^{e}\right\}}_{last}$ is the final center frequency. The value range of the rate factor *a* is 0 to 1.

Similarly, by introducing the rate factor *a* into the modal component, the initial value expression of the mode component after optimization is obtained, as shown in Formula (19).

(19) $${\left\{v_{k}^{0}\right\}}_{\mathrm{current}\;}=\left(1-a\right){\left\{v_{k}^{0}\right\}}_{\mathrm{last}\;}+a{\left\{v_{k}^{e}\right\}}_{\mathrm{last}\;}$$

where ${\left\{v_{k}^{0}\right\}}_{current}$ is the initial modal component of the current window, ${\left\{v_{k}^{0}\right\}}_{last}$ is the initial modal component of the previous sliding window, and ${\left\{v_{k}^{e}\right\}}_{last}$ is the final modal component, and the value range of rate factor *a* is also 0 to 1.

When facing complex non-steady-state signals, the unified set learning rate cannot easily deal with various upheaval situations, so relatively stable and reliable initial parameters cannot be obtained. The above findings show that when a large range of fluctuations occurs in the iteration time, the decomposition effect, such as the difference between the starting center frequency and the theoretical value at the next moment, or the similarity between the original component and the extracted modal component, will also change drastically, and these multiply. The change pattern of indicators is almost consistent, indicating that there is a real-time response relationship between iteration time and decomposition effect. Therefore, this article attempts to automatically control parameter initialization based on the fluctuations in iteration time.

The relationship between the change in time amplitude and the initial center frequency was assessed, and it was found that with a sharp increase in the iteration time, the gap between the subsequent adjacent initial center frequency and the theoretical value also increased, indicating that the center frequency of the previous window is less suitable for the current window data. In addition, the inadequacy generated increases the difficulty of the decomposition of VMD, and it takes a long time to obtain the optimal solution. At this time, if the rate factor is reduced based on the previous section algorithm, that is, when the parameters of the next window are initialized, the proportion of the center frequency of the error result is reduced, and the proportion of the initial center frequency closer to the ideal value is increased accordingly, ensuring current window rationalization of the initial parameters to avoid subsequent inefficiency of decomposition caused by improper initial values.

To summarize, this section proposes establishing a mapping relationship between the time fluctuation amplitude and rate factor, controlling the proportion of existing data participating in initialization, and conducting adaptive learning and updating of the center frequency and modal components. Through multiple experiments, we explored the relationship between the magnitude of time change, initial parameters, and rate factors, and found that there is an interval correspondence between the first two, so we constructed the correspondence relationship as in Formula (20).

(20) $$a=\left\{\begin{array}{c} 0,\Delta t\geq 0.8\\ 0.001,0.6\leq \Delta t<0.8\\ 0.01,0.4\leq \Delta t<0.6\\ 0.05,0.2\leq \Delta t<0.4\\ 0.2,0.1\leq \Delta t<0.2\\ 0.5,others \end{array}\right.$$

In the formula, Δt is the error value of the first two iteration times, and *a* is the rate factor. When the time error does not belong to the defined abnormal fluctuation range, the default value is 0.5. The algorithm first estimates the time change in real time, converts the front and back changes in the running time to the weight coefficient, and then substitutes Equations (14) and (15) to set the initial center frequency and modal components of VMD.

#### 2.2.3. Algorithm Implementation Workflow

The algorithm constructs a real-time VMD model guided by a sliding window. Firstly, it utilizes SDFT to compute the spectrum, thereby reducing the time required for frequency domain transformation. Secondly, during parameter initialization, the results from previous stages are used as the initial parameters for the current stage to reduce computational costs. Finally, a minimum absolute error criterion is added to further constrain the convergence rule, preventing potential over-decomposition phenomena during iterations.

PO-RSVMD, derived from VMD, still requires reasonable presetting of input parameters in the initial stage to ensure the accuracy of the decomposition results. Before processing the signal, the window length is selected based on the sampling characteristics of the equipment and the frequency resolution, and the sliding step size is set according to actual requirements, thereby completing the sliding window design of the algorithm. Additionally, appropriate values for the number of decomposition layers *K*, the penalty coefficient α, the maximum number of iterations $N_{max}$, the relative error $\varepsilon_{r}$, and the absolute error $\varepsilon_{a}$ are set. It is particularly noted that for the first window of data, the initial modes are set to 0, and Fourier transform is performed to obtain the spectrum $F_{current}$. The first *K* peak frequencies in the spectrum are used as the initial center frequencies. After VMD processing, the specified mode components ${\left\{u_{k}\right\}}_{current}$ and center frequencies ${\left\{\omega_{k}\right\}}_{current}$ are obtained and used as initial parameters for subsequent data, enabling recursive sliding Variational Mode Decomposition.

**Figure 1:**
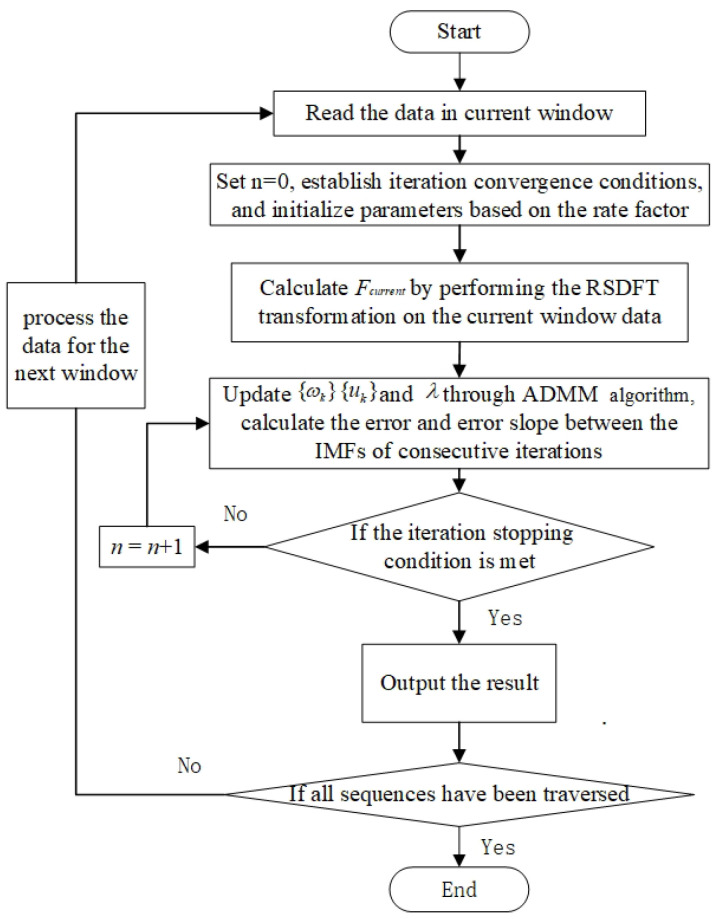
PO-RSVMD algorithm flow chart.

The flowchart of PO-RSVMD is shown in Figure 1, and the specific implementation steps are as follows.

(1) Read the current time data input value based on the pre-designed sliding window.
(2) Initialize the parameters by setting the Lagrange multiplier $\hat{\lambda }$ and the number of iterations *n* to 0. Use the decomposition results from the previous window signal to initialize the input parameters for the current window. Assign the center frequency ${\left\{\omega_{k}\right\}}_{current}$ and intrinsic modal component ${\left\{u_{k}\right\}}_{current}$ from the previous window’s output to the initial center frequency ${\left\{\omega_{k}\right\}}_{init}$ and initial modal component ${\left\{u_{k}\right\}}_{init}$ of the current window.
(3) Perform sliding discrete Fourier transform, and calculate the spectrum information of the current window using the spectrum of the previous frame data and the new and old data before and after the update, that is, Formula (7).
(4) Iterate to calculate the optimal solution; enter the internal iterative operation of VMD; solve the optimal variational decomposition of the current time data using the ADMM method; substitute the updated frequency spectrum, modal components, and central frequency under the corresponding iteration times into Formulas (8) and (9) to update the modal components and central frequency; and calculate the relative error and absolute error of this iteration according to Formulas (10) and (11).
(5) Set n=n+1. If *n* is less than the maximum number of iterations $N_{\max}$ at this time, or both the absolute and relative errors are less than the preset accuracy, then the iteration will be stopped; otherwise, repeat step (4) until the iteration stop conditions are met. Please refer to Formulas (12) and (13) for the iteration stop conditions. Perform inverse Fourier transform and output the result of this window.
(6) Slide the window with the specified step size to update the time series, and repeat steps (1) to (5) to perform Variational Mode Decomposition on the signal until all data have been processed.

## 3. Experimental Analysis and Results

The simulation experiments conducted in this study were designed to evaluate the real-time performance of PO-RSVMD in comparison to VMD and RSVMD, particularly in scenarios with strong interference. While additional simulation cases could further demonstrate the general signal decomposition capabilities of VMD-based methods, they are not directly relevant to the core objective of this study, which is to improve the computational efficiency of RSVMD for real-time applications. We implemented the PO-RSVMD algorithm using MATLAB 2021b and conducted a series of simulation experiments to evaluate its performance in comparison to VMD and RSVMD. The experiments focused on signal decomposition under varying levels of noise and interference, with a particular emphasis on real-time performance and accuracy.

### 3.1. Evaluation Metrics

The root-mean-square error (RMSE) was used to evaluate the performance metrics of the algorithm. The RMSE formula is given by Formula (21).

(21) $$\mathrm{RMSE}=\sqrt{\frac{1}{N}\sum_{n=1}^{N}{\left|x\left(n\right)-\overset{⌢}{x}\left(n\right)\right|}^{2}}$$

where x(n) is the true component of the signal, $\overset{⌢}{x}\left(n\right)$ is the component obtained after signal decomposition, and *N* is the length of the signal. The closer the decomposed signal is to the true component, the smaller the RMSE value, indicating better decomposition performance.

### 3.2. Analysis of Unstable Performance Phenomenon of RSVMD and Optimization Results

The simulation signal used in our study was sourced from reference [20], which is widely applied in various EMD and VMD simulation experiments, and in simulation experiments using their respective improved algorithms, and holds significant application and reference value, as shown in Equation (22).

(22) $$\left\{\begin{array}{c} x=x_{1}+x_{2}+x_{3}+\eta \\ x_{1}=\cos\left(2\pi \times 2t\right)\\ x_{2}=\frac{1}{4}\cos\left(2\pi \times 24t\right)\\ x_{3}=\frac{1}{16}\cos\left(2\pi \times 288t\right) \end{array}\right.$$

where *x* is the original signal, $x_{1}$, $x_{2}$, and $x_{3}$ are cosine signals with frequencies of 2 Hz, 24 Hz, and 288 Hz, respectively, and η is Gaussian white noise.

**Figure 2:**
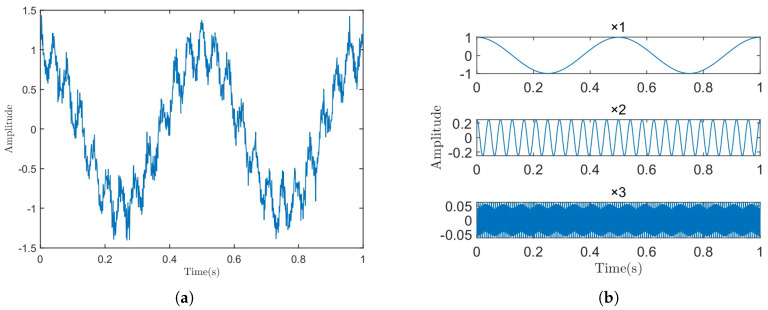
Time-domain plot of original signal and its sub-signals. (**a**) Simulation signal. (**b**) Sub-signal.

The sampling frequency of the constructed simulation signal is set to 1000 Hz, i.e., $f_{s}$ = 1000 Hz, with a sampling duration of 10 s (T = 10 s) and an SNR of 20 dB. We plotted only the first cycle of the simulation signal, as well as the time-domain waveforms of its components, as shown in Figure 2. This simulation signal is composed of three harmonic signals with specific frequencies, so the number of decomposition modes for RSVMD was set to 3. The initial center frequencies were set to the frequencies of the sub-signals, the initial mode of the first window was set to 0, the penalty factor was set to 2000, the maximum number of iterations was 500, the relative error was set to $5\times 10^{-3}$, and the iteration error was set to $5\times 10^{-6}$.

**Figure 3:**
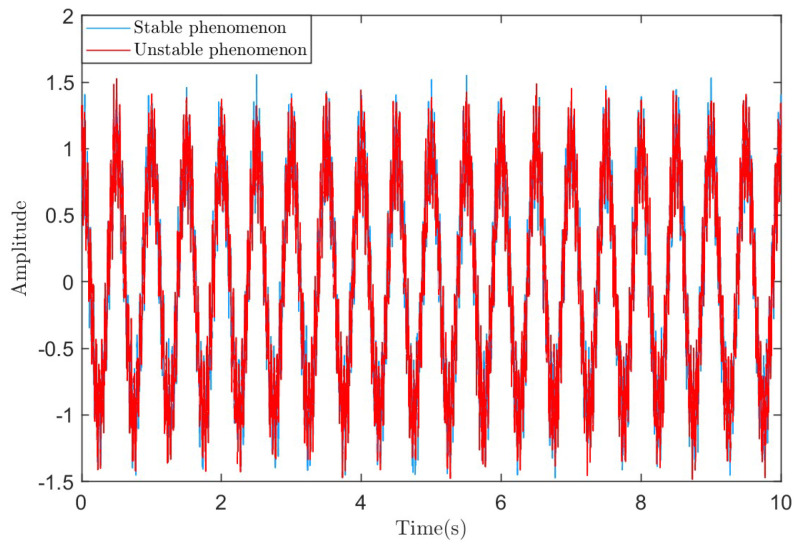
The original signals in the two phenomena.

Using [16, 1000] as the sliding step size and window length, RSVMD decomposition experiments were conducted on the noisy simulation signal described in Equation (22). After multiple experiments, it was found that in some cases, the decomposition results exhibited unstable behavior. The original signals that caused the unstable behavior were highly similar in waveform to the original signals that exhibited stable behavior, as shown in Figure 3. The difference between unstable and stable behavior phenomena in this article is that for the same signal, after adding random noise with the same signal-to-noise ratio, the overall trends and amplitudes of the resulting two signals are very similar. However, when decomposing these two highly similar signals using RSVMD with the same parameters, the decomposition results show significant differences. These differences are reflected in aspects such as the number of iterations, iteration time, center frequencies, and the discrepancies between the decomposed modes and the true signal.

**Figure 4:**
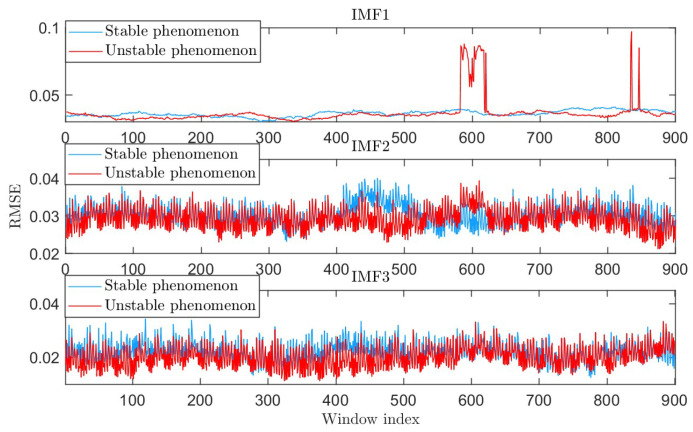
The RMSE of the sub-signals in the two phenomena.

However, under the RSVMD unstable behavior phenomenon, the error between the decomposed component IMF1 and the true component increases significantly at certain stages. We used RMSE to compare the difference in signal components between the stable and unstable behavior phenomena, as shown in Figure 4.

#### 3.2.1. Issue of Over-Decomposition

**Figure 5:**
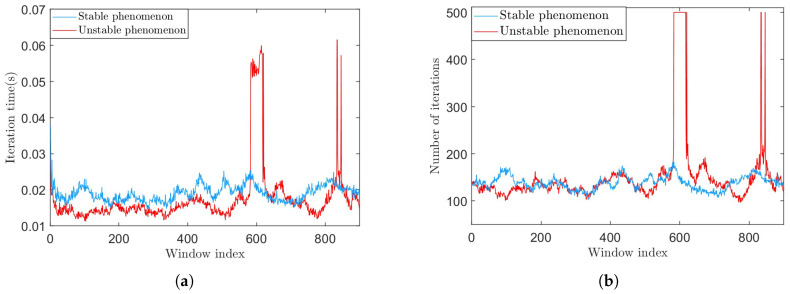
The algorithm decomposition efficiency results for the two phenomena. (**a**) The iteration time in the two phenomena. (**b**) The number of iterations in the two phenomena.

The over-decomposition phenomenon is characterized by a significant increase in the number of iterations and iteration time, as shown in Figure 5.

**Figure 6:**
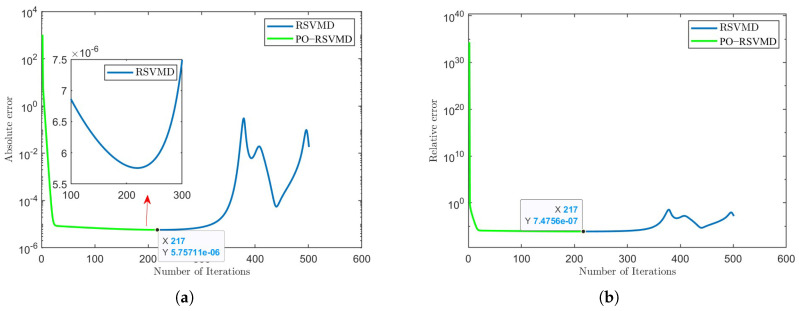
Error variation in iterative process of RSVMD and PO-RSVMD. (**a**) Variation in absolute error. (**b**) Variation in relative error.

After further adding constraints to the iteration convergence conditions for algorithm optimization, a segment of data exhibiting the unstable behavior phenomenon was used to test the effectiveness of the algorithm, as shown in Figure 6.

From Figure 6a, it can be observed that the optimized RSVMD algorithm terminates after 217 iterations. Both algorithms use the default iteration count of 500 and the default preset error parameters. From the figure, it can be observed that RSVMD exhibits over-decomposition, after which the absolute error begins to increase, and the iteration cannot be stopped. This indicates that PO-RSVMD effectively addresses the over-decomposition issue in RSVMD. To better evaluate the performance of the algorithm, we represent the modal components with IMF1, IMF2, and IMF3, corresponding to center frequencies of 288 Hz, 24 Hz, and 2 Hz, respectively, based on frequency order. The RMSE values for the two algorithms were calculated through the aforementioned optimization experiments to assess their effectiveness, as shown in Table 1. From Table 1, it can be concluded that the RMSE of each modal component in the PO-RSVMD algorithm is lower than that of the RSVMD algorithm, indicating that the algorithm’s performance has been improved.

#### 3.2.2. Issue of Increased Central Frequency Error

**Figure 7:**
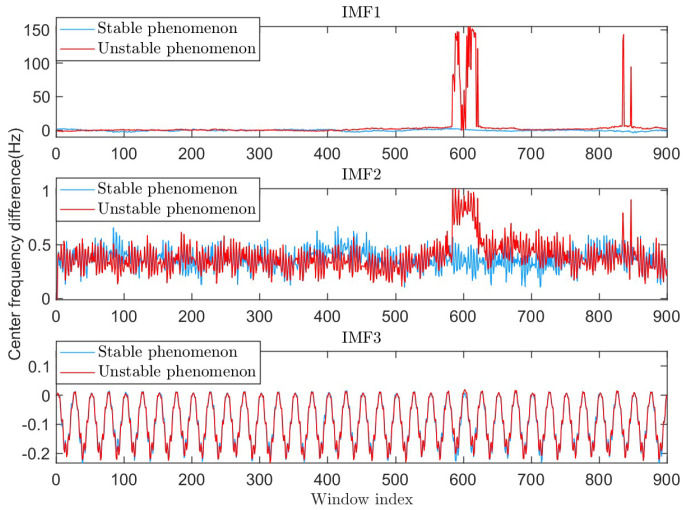
The difference between the initial center frequency and theoretical value in the two phenomena.

After the performance degradation of RSVMD, an increase in the center frequency error of each component occurs, as shown in Figure 7.

**Figure 8:**
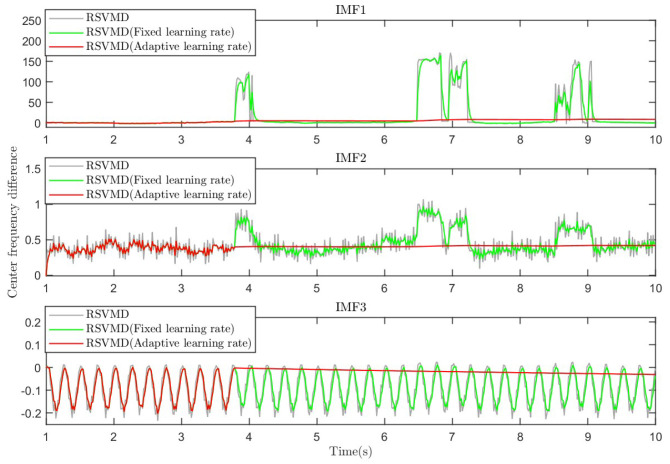
The difference between the center frequency and theoretical value in the different algorithms.

Through our research, it was found that introducing a rate learning factor can effectively reduce the error caused by the incorrect initialization of the center frequency. Introducing a fixed rate learning factor reduces the error to some extent, but when the center frequency error fluctuates significantly, the error remains unacceptable. Therefore, by automatically adjusting the rate learning factor based on the changes in iteration time, we can significantly suppress the increase in center frequency error. The experimental results are shown in Figure 8.

From Figure 8, it is evident that there is a significant discrepancy between the initial center frequency and the theoretical value in RSVMD and RSVMD with fixed rate factor initialization parameters. IMF1 exhibits several drastic fluctuations ranging from 17% to 52%, and these fluctuations persist for a relatively long duration, while the fluctuation ranges for IMF2 and IMF3 are about 5% to 10%. Although RSVMD with fixed rate factor initialization parameters mitigates the impact of errors to some extent, its effectiveness is limited. In contrast, the difference in our improved algorithm shows almost no significant fluctuations throughout the process, and at mutation points, it successfully maintains the initial center frequency near the actual value. This improvement is attributed to PO-RSVMD, which automatically adjusts the initial parameters of the previous window based on the detected changes in iteration time. This reduces the impact on the current window, effectively halts the center frequency error fluctuations, and substantially decreases the error, ensuring the accuracy of subsequent iterative operations.

### 3.3. PO-RSVMD Algorithm Performance

**Figure 9:**
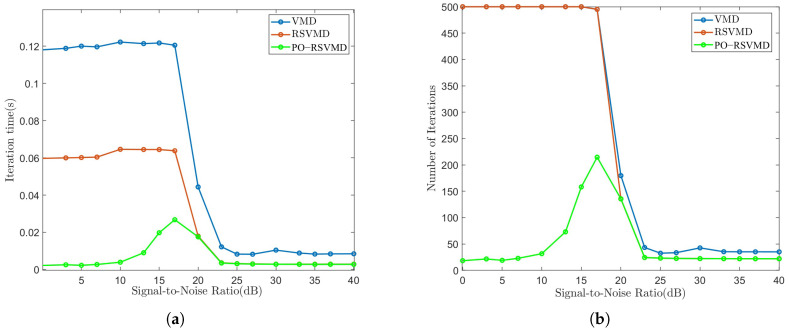
Average iteration time and average iteration count of each algorithm at different SNRs. (**a**) Average iteration time. (**b**) Average iteration count.

To make the experimental data more rigorous, we set the SNR in the range of [0, 40] with a 3 dB interval, in order to verify the real-time denoising capability of the RSVMD optimization algorithm. The experimental results are shown in Figure 9.

It is clearly seen from Figure 9a,b that the PO-RSVMD algorithm is highly efficient in terms of signal decomposition. In the [0, 17] dB SNR range, the average iteration time and iteration count for the VMD and RSVMD algorithms tend to stabilize at a fixed value, while the average iteration time and iteration count for PO-RSVMD show an increasing trend, but its averages are much smaller than those of the other two algorithms. In terms of time, PO-RSVMD is 76–97% faster than VMD and 53–95% faster than RSVMD. In terms of iteration count, PO-RSVMD reduces the number of iterations by 57–95% compared to the other two algorithms. In the [17, 40] dB SNR range, the iteration time and counts for all three algorithms show a decreasing trend, but PO-RSVMD remains the most efficient. This indicates that PO-RSVMD achieves higher decomposition efficiency than VMD and RSVMD under different SNR conditions, confirming that the algorithm can still perform real-time decomposition of useful signals even under strong interference.

**Figure 10:**
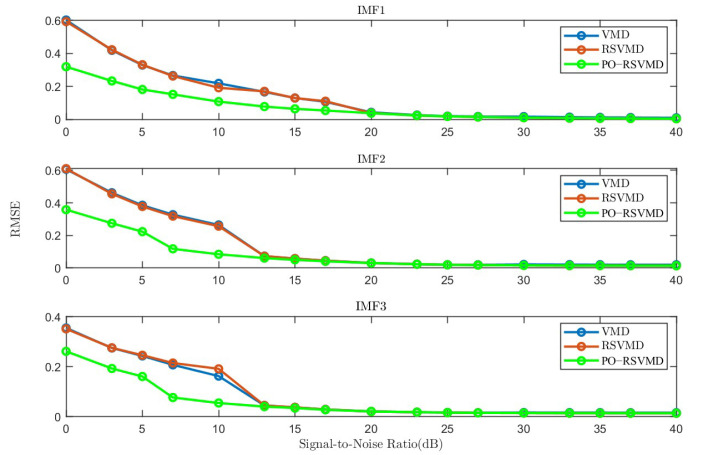
Average RMSE of three algorithms at different SNRs.

Starting from the decomposition accuracy, the average RMSE of each component derived from the three algorithms under the [0, 40] dB SNR was used, as shown in Figure 10.

As shown in Figure 10, the PO-RSVMD algorithm achieves the highest similarity between each modal component and the original component. Furthermore, in the [0, 15] dB signal-to-noise ratio range, the decomposition accuracy of this algorithm is still 35% higher than that of the other two algorithms, indicating that the PO-RSVMD algorithm has stronger denoising capabilities.

In conclusion, the PO-RSVMD algorithm can decompose signals more quickly, and its decomposition accuracy is higher than that of VMD and RSVMD, even in low signal-to-noise ratio environments. The current simulation results clearly demonstrate that PO-RSVMD significantly reduces iteration time and computational load while maintaining comparable decomposition accuracy, thereby validating its suitability for real-time signal processing in industrial polishing.

## 4. IMU Angular Velocity Denoising Experiment in Polishing Conditions

Although PO-RSVMD is an improved algorithm designed for the real-time application of VMD, its underlying principle remains fundamentally consistent with VMD in terms of denoising. As explained in Section 1, any signal can be regarded as a composite signal composed of several sub-signals with specific frequencies. Typically, useful signals are primarily concentrated in the low-frequency components, while interference signals are more prevalent in the high-frequency components. Based on this, the conventional VMD denoising strategy involves removing disturbance components according to relevant signal indicators and then superimposing the retained signal components to reconstruct a more accurate signal. Following this approach, we similarly consider the low-frequency components obtained after removing the high-frequency components as the denoised signal.

VMD denoising involves two key stages, signal decomposition and component reconstruction, with the effectiveness of denoising closely related to the decomposition parameters and the reconstruction parameters. In the decomposition stage, the main parameters include the number of decomposition modes and the penalty factor. In the reconstruction stage, the parameters refer to the number of modal components retained. During the decomposition stage, if the number of decomposition modes is too high or the penalty factor is too large, over-decomposition can occur, leading to the emergence of spurious components. Conversely, if the number of decomposition modes is too low or the penalty factor is too small, modal mixing will occur, causing a decrease in decomposition accuracy. In the reconstruction stage, the reconstruction coefficient, i.e., the number of modal components retained, plays a critical role. Retaining too many modal components makes it difficult to effectively filter out interference components, while retaining too few may result in the removal of useful signals along with noise. Therefore, the choice of these two types of parameters will significantly affect subsequent quantitative analysis and modeling work.

**Figure 11:**
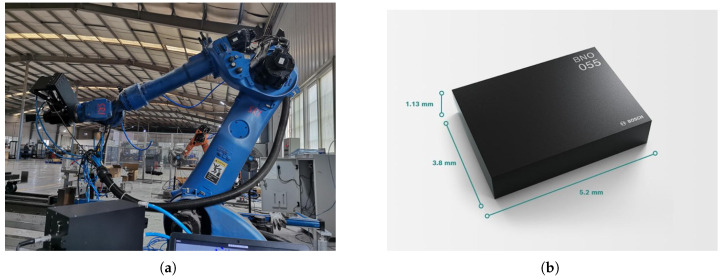
A field operation diagram of the industrial robot and the model of the IMU used. (**a**) Industrial robot. (**b**) BNO005.

In industrial polishing operations, the polishing head is subjected to severe interference from strong vibrations, impacts, and high-speed rotations, which causes the IMU sensor’s output signal to be mixed with a large number of interference components, rendering the IMU unable to function properly. In terms of application analysis, the data were collected from an IMU chip mounted on an industrial robotic arm. The industrial robot was a KUKA robot, equipped with an IMU with chip model BNO005, which is an industrial-grade IMU capable of outputting three-axis acceleration and angular velocity information. The signal sampling frequency was 50 Hz. Figure 11a shows a schematic diagram of the on-site polishing robotic arm, and Figure 11b provides the model and dimensions of the IMU chip.

**Figure 12:**
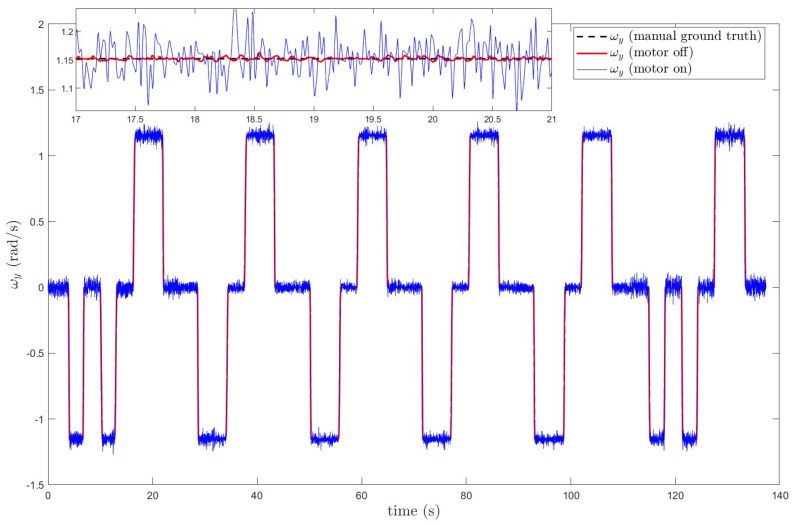
Y-axis angular velocity before and after motor operation.

Before the polishing motor starts, the IMU’s angular velocity is relatively smooth and stable. However, after the motor starts, mechanical vibrations cause the IMU’s angular velocity to be contaminated with significant disturbance signals. As shown in Figure 12.

**Figure 13:**
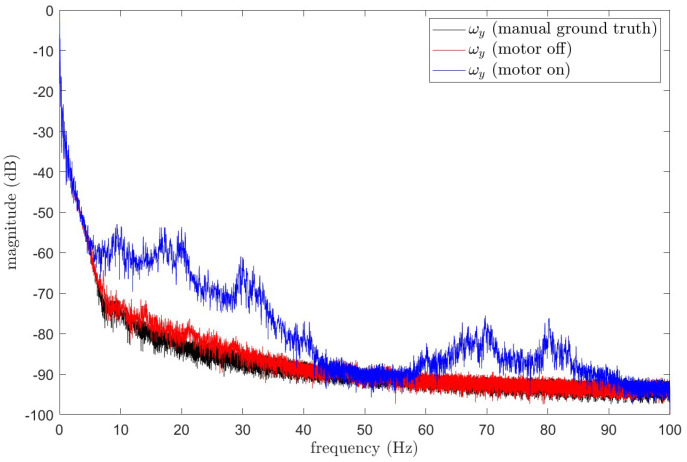
Frequency component analysis of raw data.

Figure 12 illustrates the variation in the robotic arm’s Y-axis angular velocity before and after the operation of the polishing motor. Since the robotic arm still exhibits slight vibrations even when the motor is not operating, causing minor oscillations in the IMU’s angular velocity output, the average angular velocity from multiple experiments before motor operation is taken as the ground truth for comparison. From the figure, it is evident that motor operation significantly interferes with the IMU’s angular velocity output. To more clearly identify the main frequency components of the vibration interference, the Welch power spectral density estimation method was applied to perform a spectral analysis of the raw data in Figure 12. The results are shown in Figure 13.

**Figure 14:**
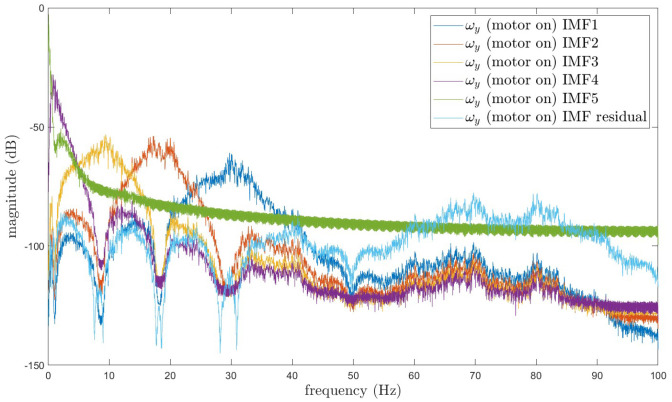
PO-RSVMD decomposition angular velocity sampling data.

The results of decomposing the angular velocity sampling data using the PO-RSVMD method are presented. The figure displays the frequency spectra of each IMF (Intrinsic Mode Function) component and compares them with the spectral components in Figure 13. The results demonstrate that PO-RSVMD can effectively separate the vibration interference, validating the effectiveness of PO-RSVMD in this application, as shown in Figure 14.

In order to verify the high real-time performance and stability of PO-RSVMD in this polishing project scenario, comparisons of the center frequency variation and iteration times between RSVMD and PO-RSVMD and the RMSE results were carried out. We compared PO-RSVMD with RSVMD based on this experiment and found that PO-RSVMD significantly reduces the number of iterations, shortens the runtime, and still maintains a high level of decomposition accuracy. The relevant experimental parameters were set as follows: the RSVMD window length was 40 s, and the window step size was 0.1 s. The update rate in Formula (18) was set to 0.1/40 = 0.0025. The $\omega_{y}$ data from the motor startup experiment were selected for analysis.

**Figure 15:**
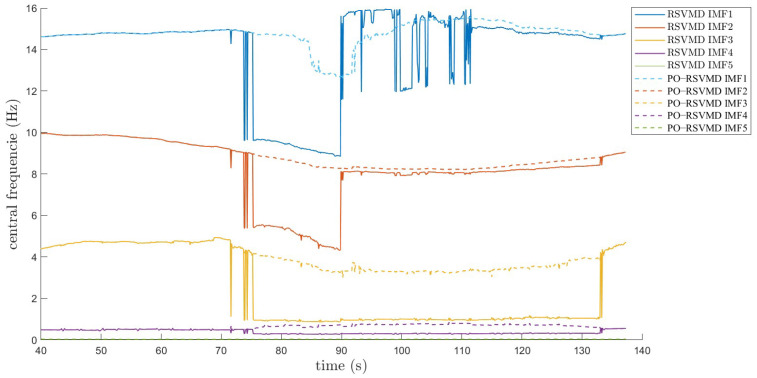
Comparison of center frequency variation between RSVMD and PO-RSVMD.

There is a significant difference in the variation in center frequencies between RSVMD and PO-RSVMD, as shown in Figure 15. During the real-time window iteration process, even though only a small amount of data was updated each time (e.g., with a window step size of 0.1 s), the center frequency of RSVMD exhibits substantial fluctuations. These large fluctuations may lead to instability in the decomposition results, particularly when dealing with high-frequency vibration interference, where the drastic changes in center frequency can affect the accuracy and reliability of the algorithm. The proposed PO-RSVMD in this paper effectively mitigates the severe fluctuations in center frequency by introducing a learning and updating mechanism for the initial frequency. PO-RSVMD can more stably adjust the center frequency during each window update, thereby reducing frequency oscillations and enhancing the robustness of the algorithm.

**Figure 16:**
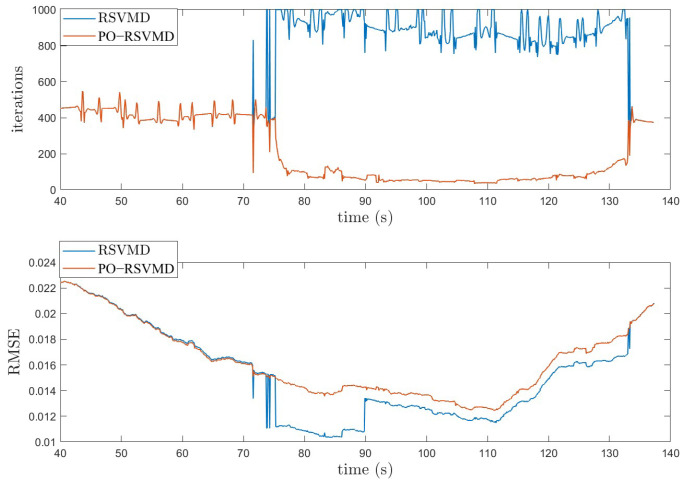
RSVMD and PO-RSVMD iterations and RMSE comparison graph.

As shown in Figure 16, it can be observed that the number of iterations for RSVMD increases dramatically during the time period from 73 to 133 s, while the number of iterations for PO-RSVMD is significantly reduced, and its RMSE is very close to that of RSVMD. This high number of iterations not only increases the computational complexity of the algorithm, but also leads to a longer runtime. Especially when dealing with high-frequency vibration interference, an increase in the number of iterations significantly affects the real-time performance of the algorithm. PO-RSVMD still maintains a low RMSE value while reducing the number of iterations. This improvement allows PO-RSVMD to have higher real-time performance without performance degradation in practical applications.

In order to evaluate the performance differences between the PO-RSVMD and RSVMD algorithms, this paper compares the average number of iterations, average iteration time, and average RMSE values of the two algorithms. See Table 2 for the specific comparison results.

It can be found in Table 2 that the average number of iterations and time of PO-RSVMD in this experiment are significantly lower than those of RSVMD, which shows that it can quickly process IMU signals in the motor polishing scenario, and the average RMSE difference between the two algorithms is very small, which verifies that The PO-RSVMD algorithm has high accuracy and good robustness in real-time applications.

## 5. Conclusions

This paper proposes the PO-RSVMD algorithm to address the real-time performance limitations of RSVMD in industrial polishing applications. During the exploration of RSVMD applications, we identified two performance degradation phenomena in practical use: over-decomposition and increased center frequency error. To address these issues, the former was optimized by introducing iterative termination conditions to detect and handle component error mutations, while the latter was mitigated by further incorporating an adaptive rate learning factor to adjust the initialization of center frequency parameters. Simulation experiments verified that PO-RSVMD outperforms VMD and RSVMD with faster iteration times, fewer iterations, and significantly lower RMSE values for the decomposed modal components, indicating higher decomposition efficiency. Finally, we applied PO-RSVMD and RSVMD to denoising experiments performed by IMU signals under strong interference conditions. The results show that PO-RSVMD requires much less iteration time than RSVMD, and both can effectively decompose signals while ensuring their accuracy. The primary contribution of this work lies in the significant reduction in computational complexity and iteration time while maintaining comparable decomposition accuracy. Although the algorithm’s performance in handling arbitrary amplitude or frequency modulation signals is an important direction for future research, the current study focuses on optimizing RSVMD for real-time applications, particularly in the context of IMU signal processing. Future work will explore the applicability of PO-RSVMD to a broader range of signal types and further enhance its robustness in complex signal decomposition scenarios.

## Figures and Tables

**Table 1:** RMSE of sub-signals for the two algorithms.

Algorithm	IMF1	IMF2	IMF3
RSVMD	0.867	0.0387	0.0281
PO-RSVMD	0.0391	0.0362	0.0273

**Table 2:** Iteration count, iteration time, and RMSE value of RSVMD and PO-RSVMD.

Algorithm	Average Iteration Count	Average Iteration Time (s)	Average RMSE
PO-RSVMD	22	0.029	0.022
	23	0.031	0.022
	20	0.028	0.018
	35	0.049	0.031
	25	0.035	0.021
RSVMD	1262	1.710	0.014
	1252	1.700	0.014
	1216	1.640	0.014
	919	1.259	0.024
	1230	1.671	0.013

## Data Availability

Publicly available datasets were analyzed in this study.
